# Bacterial Biomarkers of Marcellus Shale Activity in Pennsylvania

**DOI:** 10.3389/fmicb.2018.01697

**Published:** 2018-08-02

**Authors:** Jeremy R. Chen See, Nikea Ulrich, Hephzibah Nwanosike, Christopher J. McLimans, Vasily Tokarev, Justin R. Wright, Maria F. Campa, Christopher J. Grant, Terry C. Hazen, Jonathan M. Niles, Daniel Ressler, Regina Lamendella

**Affiliations:** ^1^Department of Biology, Juniata College, Huntingdon, PA, United States; ^2^The Bredesen Center, The University of Tennessee, Knoxville, Knoxville, TN, United States; ^3^Department of Civil and Environmental Engineering, The University of Tennessee, Knoxville, Knoxville, TN, United States; ^4^Biosciences Division, Oak Ridge National Laboratory, Oak Ridge, TN, United States; ^5^Freshwater Research Initiative, Susquehanna University, Selinsgrove, PA, United States; ^6^Department of Earth and Environmental Sciences, Susquehanna University, Selinsgrove, PA, United States

**Keywords:** hydraulic fracturing, fracking, Marcellus shale, 16S rRNA gene sequencing, microbial communities, biomarkers, halophilic, hydrocarbons

## Abstract

Unconventional oil and gas (UOG) extraction, also known as hydraulic fracturing, is becoming more prevalent with the increasing use and demand for natural gas; however, the full extent of its environmental impacts is still unknown. Here we measured physicochemical properties and bacterial community composition of sediment samples taken from twenty-eight streams within the Marcellus shale formation in northeastern Pennsylvania differentially impacted by hydraulic fracturing activities. Fourteen of the streams were classified as UOG+, and thirteen were classified as UOG- based on the presence of UOG extraction in their respective watersheds. One stream was located in a watershed that previously had UOG extraction activities but was recently abandoned. We utilized high-throughput sequencing of the 16S rRNA gene to infer differences in sediment aquatic bacterial community structure between UOG+ and UOG- streams, as well as correlate bacterial community structure to physicochemical water parameters. Although overall alpha and beta diversity differences were not observed, there were a plethora of significantly enriched operational taxonomic units (OTUs) within UOG+ and UOG- samples. Our biomarker analysis revealed many of the bacterial taxa enriched in UOG+ streams can live in saline conditions, such as Rubrobacteraceae. In addition, several bacterial taxa capable of hydrocarbon degradation were also enriched in UOG+ samples, including Oceanospirillaceae. Methanotrophic taxa, such as Methylococcales, were significantly enriched as well. Several taxa that were identified as enriched in these samples were enriched in samples taken from different streams in 2014; moreover, partial least squares discriminant analysis (PLS-DA) revealed clustering between streams from the different studies based on the presence of hydraulic fracturing along the second axis. This study revealed significant differences between bacterial assemblages within stream sediments of UOG+ and UOG- streams and identified several potential biomarkers for evaluating and monitoring the response of autochthonous bacterial communities to potential hydraulic fracturing impacts.

## Introduction

Over the past several years, the rapid increased use of natural gas in the United States (Lieskovsky et al., 2014; Laurenzi et al., 2016; U.S. EIA, 2017a) has been driven by horizontal drilling and hydraulic fracturing (Brittingham et al., 2014; Jackson et al., 2014; Burden et al., 2016). During hydraulic fracturing, a mixture of water, proppants, and chemicals is injected into a rock formation to create fractures, allowing natural gas to flow to the surface (Jackson et al., 2014; Silva et al., 2014). Hydraulic fracturing will become more prevalent as the use and demand for natural gas increases. By 2050, the use of natural gas is predicted to increase more than any other fuel source in the United States (U.S. EIA, 2017a). In the EIA’s reference case, natural gas consumption increases by 6.4 quadrillion BTUs to 32.27 quadrillion BTUs from 2015 to 2050 in the United States (U.S. EIA, 2017a), and by 90.8 quadrillion BTUs from 2014 to 2050 to 218.2 quadrillion BTUs globally (U.S. EIA, 2017b). In the United States, the Marcellus and Utica formations are predicted to be the primary drivers of this growth (U.S. EIA, 2017a).

Technological advances in natural gas extraction have far outpaced our ability to evaluate the potential environmental impacts of this process. Recent research has indicated possible contamination of water resources by nascent hydraulic fracturing activities (Rahm and Riha, 2012; Jackson et al., 2013; DiGiulio and Jackson, 2016). Hydraulic fracturing produces a large amount of saline wastewater (Veil et al., 2004; Cluff et al., 2014; Silva et al., 2014); wastewater can enter nearby groundwater or surface water via faulty well casings, leaks in holding ponds, or spills (Vidic et al., 2013). This could be especially problematic as high salinities can inhibit the biodegradation of potentially harmful chemicals in hydraulic fracturing fluid (Kekacs et al., 2015), several of which have been found in produced water (Burden et al., 2016). Furthermore, there have been over 4,000 violations of environmental health and safety regulations for hydraulic fracturing operations in Pennsylvania since 2009 (PA DEP, 2017a). Additionally, changes in land use can also impact adjacent streams, as each well pad needs between 1.2 and 2.7 acres of land on average (Brittingham et al., 2014). Increased overland flow due to forest disturbance has been found to affect water quality (Schelker et al., 2012; Palviainen et al., 2014). Undoubtedly then, hydraulic fracturing has the potential to affect nearby water resources. This issue is especially pertinent to Pennsylvania, which overlies the Marcellus shale formation and has around 10,000 active hydraulic fracturing wells (PA DEP, 2017b). Furthermore, it is currently the second largest producer of natural gas in the United States (U.S. EIA, 2017c).

Stream ecosystems are particularly sensitive to disturbances within their watershed (Allan, 2004; Luke et al., 2017) and can impact both local and regional water quality and biodiversity (Meyer et al., 2007). Therefore, it is important to understand how hydraulic fracturing, unconventional oil and gas (UOG) extraction, impacts nearby streams. Sediments in streams are especially important to examine due to their ability to accumulate pollutants, making them excellent long-term indicators of stream health (Sekabira et al., 2010; Schmeller et al., 2018). Within sediments, microbial communities can serve as further indicators of stream quality, as microbes can quickly respond to disturbances (Hunt and Ward, 2015). In addition, microbes are the most abundant organisms in aquatic communities, by concentration and often even by biomass (Hunt and Ward, 2015). As a result, they play an important role in cycling energy and nutrients. Microbial communities have been found to change in response to anthropogenic activities, such as agriculture, industry, and mining (Wassel and Mills, 1983; Essahale et al., 2010; Stutter and Cains, 2017).

Recent work has found streams near hydraulic fracturing experience drastic shifts in certain bacterial assemblages (Acetobacteraceae, Methylocystaceae, and *Phenylobacterium*) and had lower observed bacterial diversity (Trexler et al., 2014). However, a study done in the Fayetteville shale formation did not find any significant difference in alpha diversity based on proximity to hydraulic fracturing, but it too noted an increased abundance of specific taxa (*Microcystis* and Synechoccophycideae) in streams near hydraulic fracturing operations (Johnson et al., 2017). Furthermore, that study noted little work has been done on evaluating the impact of hydraulic fracturing on streams. This is especially surprising considering their importance and sensitivity to environmental disturbances. More recently, specific bacteria, including the aptly named *Frackibacter*, have been found to be associated with hydraulic fracturing (Daly et al., 2016; Mouser et al., 2016). Here we utilized bacterial community profiling to address the aquatic bacterial response (*n* = 31 streams) to potential hydraulic fracturing inputs in Pennsylvania, which is experiencing the most rapid development in UOG in the United States (U.S. EIA, 2016). We hypothesized that the alpha and beta diversity of UOG+ and UOG- streams would differ significantly and certain OTUs and pathways would be enriched based on proximity to hydraulic fracturing. High-throughput sequencing of the 16S rRNA gene and random forest modeling enabled the identification of several bacterial taxa that were enriched and predictive of UOG status of a given stream. The data generated in this study can potentially be used and combined with future aquatic microbial ecology studies to generate more comprehensive spatial and temporal biomarkers of UOG activity.

## Materials and Methods

### Site Description and Sampling

**FIGURE 1:**
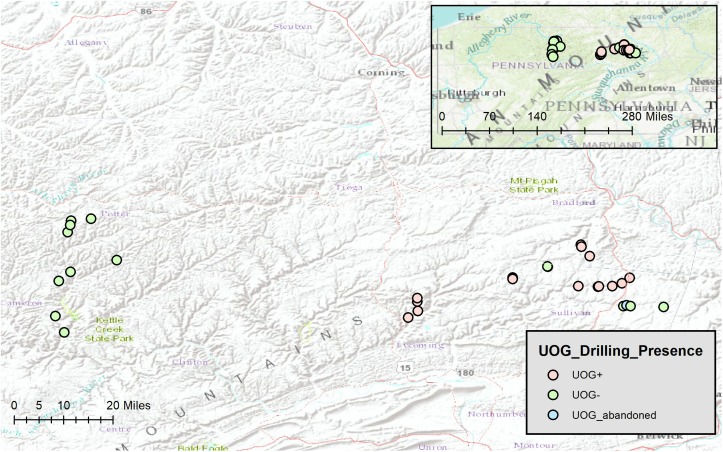
Map of Stream Sites made with ArcGIS (Esri, 2016). Stream sites (n = 31) were located across Bradford, Cameron, Lycoming, Potter, and Sullivan counties of northeastern Pennsylvania. Red represents UOG+, green represents UOG-, and blue represents UOG_abandoned.

Sediment samples were taken from thirty-one streams in northeastern Pennsylvania (**Figure 1**) during the summer of 2016. Of these, fourteen were classified as UOG- (no well pads with active wells present in the watershed) sixteen were classified as UOG+ (at least one well pad with at least one active well present in the watershed), and one was classified as UOG abandoned (no UOG activity in the watershed within two and a half years prior to sampling); all wells present in the watershed (active, plugged, and regulatory inactive) were included in the well count number for UOG+ streams and the UOG abandoned stream (Supplementary Table 1).

Site selection of northeastern PA streams was done from a larger sub-set of potential streams using ArcGis 10.4 (Esri). Watershed boundaries were delineated using the watershed pour method to determine the size and boundary of the watershed from the sample site location. UOG gas wells’ layers and information were obtained from PA DEP (PASDA) and overlaid onto watersheds. Google Earth imagery was used to verify existence of well pads in each watershed. Because DEP data only gave a singular point for each well and not a polygon, well pads were then digitized and overlaid into the watershed layer to create a polygon of well pads. If any portion of a well pad was in the watershed then it was considered part of the watershed due to the likelihood of spills and contaminants spilling across that well pad area. Final sampling sites were determined based upon presence of UOG gas wells and ability for access (either public lands or permission obtained from private landowners). In order to determine that sample sites were comparable site specific information was analyzed in ArcGis 10.4 to determine in which EPA ecoregion sample sites were located. In addition, a detailed land use analysis was performed for each watershed in ArcGis 10.4 using the National Land Cover Database (Homer et al., 2015) to determine if there were differences among land use in UOG- and UOG+ sample sites.

All sampling sites were located in valleys in the North Central Appalachian or the Northern Allegheny Plateau ecoregion (Supplementary Table 2). None of the streams were in state parks. Land use and % vegetation composition were determined for our sampling sites (Supplementary Table 2). Sites were determined to be comparable stream order as well, with most streams being first order (Supplementary Table 2). Water quality (alkalinity, conductivity, pH, and temperature) was also measured (Supplementary Table 1). Because the data were not normal, Kruskal–Wallis and Wilcoxon Rank Sum tests were used to determine if those factors differed significantly between UOG+ and UOG- streams. Of the factors tested, only percent of woody wetlands vegetation differed significantly (*p* < 0.05) between UOG+ and UOG- streams (Supplementary Table 3).

Two fine sediment samples were collected from each stream as described in Trexler et al. (2014). Briefly, for each stream, a sterile scoop was used to collect a fine sediment sample. Fine sediment was collected at a depth of 0–1 cm from pool habitat (from either main channel or side channel pools), and subsequently placed in a sterile 50 mL conical tube, before being stored on ice in a cooler until the samples were returned to the laboratory, where they were stored at -80°C. A YSI Pro Plus probe was used to measure conductivity, pH, and temperature at each sampling site. Alkalinity was measured by field titrating using standard methods. Water quality parameters were measured to evaluate abiotic conditions in streams at the time of sampling. Because water and sediments in streams are exposed to similar conditions, long term trends in water quality parameters should parallel those in sediments, as previously described (Bauch et al., 2009; McDaniel et al., 2009; Reed and Martiny, 2013).

### DNA Extraction and 16S rRNA Library Preparation

DNA extractions were performed on the sediment samples (*n* = 31) using a Mo Bio PowerSoil DNA Isolation Kit according to the manufacturer’s instructions (QIAGEN, Germantown, MD, United States). The resulting extracts were quantified with Qubit 2.0 fluorometer double-stranded DNA (dsDNA) high sensitivity DNA kit according to the manufacturer’s instructions (Invitrogen, Carlsbad, CA, United States) and then stored in a -80°C freezer.

Illumina tag PCR mixtures for each sample consisted of 2.5 μL of 10x PCR buffer, 0.8 mM of dinucleoside triphosphates (dNTPs), 0.2 μM of 515F forward primer, 0.2 μM of Illumina 806R reverse barcoded primer, 0.625 U of *Taq* polymerase, 25 μM of Betaine, and various amounts of DNA to optimize yield for a total volume of 25 μL per sample. The reactions were run on a MJ Research PTC-200 thermocycler (Bio-Rad, Hercules, CA, United States), with the conditions being 94°C for 3 min., then 95 cycles of 94°C for 45 s, 53°C for 60 s, and 72°C for 90 s, terminating at 72°C for 4 min. and then holding at 4°C. The PCR products were checked on a 2% agarose safe E-Gel that was pre-stained with SYBR (Invitrogen, Carlsbad, CA, United States) for PCR products of target length (∼390 bp). PCR products were pooled in equimolar ratios and were gel purified using a 2% agarose gel and a QIAquick Gel Extraction Kit per the manufacturer’s protocols (QIAGEN, Germantown, MD, United States).

The purified libraries were then analyzed using an Agilent Bioanalyzer (Agilent, Santa Clara, CA, United States). Afterwards, the libraries were stored at -20°C until they were shipped on dry ice to California State University (Los Angeles, CA, United States) where they were sequenced on the Illumina Miseq with v2 300 cycle chemistry (Illumina, San Diego, CA, United States) to produce 250 bp paired-end reads.

### Bioinformatics and Statistical Analysis

A total of 31 samples were successfully sequenced, encompassing 3,519,171 reads. The raw sequence data that were used for analysis can be found in NCBI’s Short Read Archive under accession number SRP139372. Due to the poor quality of the reverse reads, forward and reverse reads could not be adequately paired, and analysis was done using only forward reads (average Q-score = 34.6), as this method has been shown to produce robust taxonomic classification (Liu et al., 2007; Caporaso et al., 2011). Sequences were filtered for quality with a max expected error of 0.500 and a truncation length of 246 bp, using USEARCH version 7 (Edgar, 2010). QIIME 1.9.1 (Caporaso et al., 2010) was then used to analyze the filtered sequences. Operational taxonomic units (OTUs) were determined using the UPARSE algorithm *de novo* method, with sequences that were 97% similar being classified as an OTU. Data analyses mainly focused on taxa at the family level due to an inability to classify many OTUs at the genus level (Supplementary Figure 1). Taxonomy was assigned using the Greengenes 16S rRNA database (13–8 release). Singletons were removed and three samples were discarded due to having less than 1,000 sequences; the remaining 28 samples (13 UOG-, 14 UOG+, 1 UOG abandoned) all had at least 21,000 sequences, comprising 17,308 OTUs and 2,402,378 sequences.

Alpha diversity was analyzed with QIIME 1.9.1, using an OTU table rarefied to a minimum depth of 200 sequences and a maximum of 21,590 sequences with a step size of 100 and 20 iterations. The alpha diversity metrics used included Chao1, Heip’s evenness, observed, and phylogenetic distance (PD) whole tree species richness metrics to investigate differences in alpha diversity between UOG+ and UOG- samples. A nonparametric two-sample *t*-test was conducted to compare sample types, using 999 Monte Carlo permutations.

Weighted UniFrac distances were determined using the OTU table after it had undergone cumulative sum scaling (CSS) normalization to show beta diversity. A principle component analysis (PCoA) Emperor plot (Vázquez-Baeza et al., 2013) was created with QIIME to examine potential clustering among the samples based on UOG drilling presence, wells, and water quality measurements (alkalinity, conductivity, pH, and temperature), using a weighted UniFrac distance matrix. This analysis was performed to determine if samples clustered similarly based on proximity to hydraulic fracturing as they did with water quality measurements expected to differ based on proximity to hydraulic fracturing, namely conductivity and pH. ANOSIM and PERMANOVA tests were performed on the weighted UniFrac distance matrix to see if samples differed significantly based on UOG drilling presence, UOG+ or UOG-. Adonis tests were performed on the same matrix as well to measure how much variation could be explained by UOG drilling presence, number of wells, and water quality measurements. Kruskal–Wallis and Wilcoxon rank sum tests were used to detect significant differences in water quality data, which were not normally distributed. All statistical tests were considered significant at α = 0.05.

Linear discriminant analysis (LDA) effect size, LEfSe, (Segata et al., 2011) was performed to see if any OTUs were enriched based on UOG drilling status. A Kruskal–Wallis test and pairwise Wilcoxon rank sum test correction were used to determine if OTUs differed significantly in abundance between UOG+ and UOG- samples using relative abundances from the CSS normalized OTU table at the family level. Both tests were performed with α = 0.05. LDA was then used to determine the effect sizes of the enriched OTUs. A LEfSe plot and cladogram were created to show the results.

Random forest modeling was done with R (R Core Team, 2017) using CSS normalized relative abundances of enriched families to see how accurately UOG+ and UOG- sites could be classified based on those enriched OTUs. The caret package (Kuhn, 2017) was used to partition 80% of the data for a training set and the remainder for a test set. It also decided how many OTUs were considered at each step. The random forest package (Liaw et al., 2002) was then used to create a list of predictors and their GINI decreases based on 66% of the training set data, with the remainder of that data being used for an out-of-bag error estimate. Those predictors were used to classify the sites in the test set as UOG+ or UOG- to validate the model. After repeating this process 1,000 times, the percent of sites correctly classified were averaged to see how well the random forest model performed, and the OTUs’ GINI decreases were averaged to see which OTUs were most important for classifying sites. A GINI score is a measure of impurity, with higher scores reflecting greater group impurity. Accordingly, the decreases in GINI score, denote the strength with which a given OTU could differentiate UOG+ samples from UOG-.

Two co-occurrence networks were created for UOG+ and UOG- sites using Cytoscape 3.3.0 (Shannon et al., 2003) with the CoNet plugin (Faust and Raes, 2016) to examine correlations among OTUs in sediment samples. OTU tables showing relative abundances at the genus level were used. OTUs were only considered if they appeared in at least half of the sampling sites (*n* = 7), were identified to at least class and had a Spearman’s rho of at least 0.9. Spearman correlation, Bray–Curtis dissimilarity, and Kullback–Leibler dissimilarity were used for determining correlations among the taxa. Benjamin–Hochberg multiple test correction was used to adjust *p*-values and Brown’s method was used to merge *p*-values in cases where multiple edges connected the same pair of nodes. The networks were then juxtaposed and compared using CytoGEDEVO (Malek et al., 2016). Nodes for the same OTUs were matched with a null pairing penalty of 0, revealing which OTUs were present in both networks and which were exclusive to one. Nodes were then moved so that labels would not overlap. Nodes with only one edge were removed from the final network as they did not meaningfully impact the overall networks (See Supplementary Figure 2 for the original combined network).

PICRUSt analysis (Huttenhower et al., 2013) was done on a closed-reference OTU table, made using QIIME 1.9.1 and normalized by copy number, to predict the functional capabilities of the bacteria in our samples. OTUs were determined based on 97% similarity. RDP classifier was used to assign taxonomy (Wang et al., 2007) based on the Greengenes 16S rRNA database (13–8 release). Significant differences in functional pathway abundance between UOG+ and UOG- samples were determined using LEfSe analysis as previously described, except the LDA threshold was lowered to 1. The results were then plotted.

Partial least squares discriminant analysis (PLS-DA) was performed within R using the mixOmics package (Cao et al., 2017) to compare aquatic microbial communities in this study (northeastern PA) to previously collected stream sediments in 2014 (central PA) (Supplementary Figure 3). Samples from the previous study were classified as HF+ or HF-, based on proximity to hydraulic fracturing. Sequence data from sediment samples from Ulrich et al. (2018) and this study were merged and quality filtered at an average expected error of 0.5 and truncation length of 150 bp. All downstream steps for filtering, OTU clustering, and normalization were performed as described above. Data were fitted to three components to generate the PLS-DA model (Supplementary Figure 4). The model was assessed with 10-fold cross-validation repeated ten times.

## Results

### Stream Parameters

None of the water quality measures differed significantly between UOG+ and UOG- streams (Kruskal–Wallis and Wilcoxon rank sum tests *p* > 0.05). Additionally, none of the land use or ecological features we measured differed significantly, except for percent of woody wetland vegetation (Supplementary Table 3). However, percent of woody wetland vegetation did not explain a significant amount of variation among our bacterial communities (Adonis, *p* > 0.05). The number of wells in UOG+ watersheds ranged from 1 to 22. Chemical and physical parameters collected (Alkalinity, Conductivity, pH, and Temperature) had a relatively weak correlation with bacterial community structure (BIOENV, *R* = 0.2064). Of the measured parameters, temperature and pH (α = 0.05) explained the most variation in bacterial community composition (Adonis, *R*^2^ = 0.0977 and 0.0650 respectively). Additionally, pH appears to be associated in part with geography (HUC10 watershed) (Kruskal–Wallis, *p* = 0.0110), but HUC10 watershed did not significantly explain differences among the samples’ bacterial communities (adonis and PERMANOVA, *p* > 0.05, and Supplementary Figure 5). HUC8 watershed and the EPA ecoregion classifications also did not significantly explain differences among the samples’ bacterial communities (adonis and PERMANOVA, *p* > 0.05).

### Bacterial Community Diversity

On average, stream sediment samples yielded 85,799 sequences per sample after quality filtering. UOG+ samples had an average of 3,442 ± 661 OTUs, and UOG- samples had an average of 3,121 ± 807 OTUs. The UOG abandoned sample had 3710 OTUs. None of the measures of alpha diversity (Chao1, Heip’s evenness, observed, and PD whole tree) were found to differ significantly among the sampling sites based on UOG drilling presence (*p* > 0.05). Beta diversity analyses also revealed that samples did not cluster based on UOG drilling presence, nor was there a significant difference between UOG+ and UOG- communities (Adonis, *p* = 0.259; ANOSIM, *p* = 0.2970; PERMANOVA, *p* = 0.2920). While we only obtained one UOG abandoned sample, it did not appear to meaningfully differ from the UOG+ and UOG- samples with respect to beta diversity or taxonomic composition, as it falls near several UOG+ and UOG- samples within the beta diversity plots (Supplementary Figure 6). Number of wells within a watershed also did not significantly explain variation between samples (Adonis, *p* = 0.207). Therefore, UOG drilling presence does not appear to have significantly affected the overall alpha or beta diversity of sediment bacterial communities from streams sampled in this study.

**FIGURE 2:**
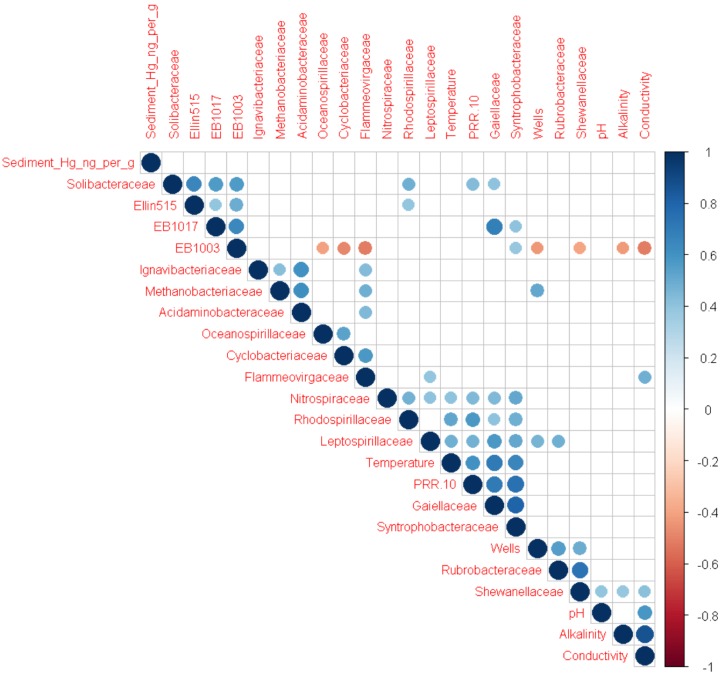
Correlogram showing significant Spearman correlations (α = 0.05) among metadata parameters and bacterial families enriched based on UOG drilling presence. Color indicates whether the correlation is positive (blue) or negative (red). Size and darkness of the circles indicate the strength of the correlations, with stronger correlations being larger and darker than weaker ones. The R packages Hmisc (Harrell, 2017) and corrplot (Wei and Simko, 2017) were used to calculate Spearman correlations and generate the correlogram.

**FIGURE 3:**
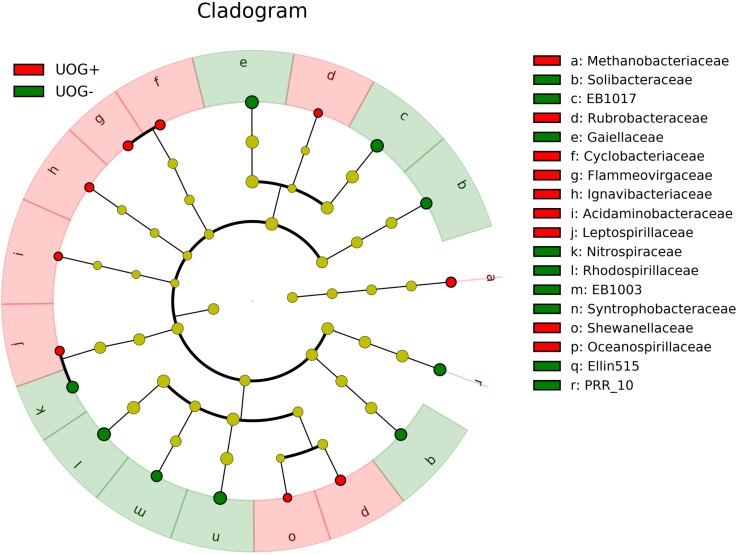
Cladogram showing differentially enriched families between UOG+ and UOG– samples. UOG+ enriched taxa are indicated in red, and UOG– enriched taxa are indicated in green. A Kruskal–Wallis test was used to identify enriched taxa (α = 0.05). Effect sizes for the enriched taxa were determined using linear discriminant analysis (LDA). Taxa with LDA scores ≥2 are shown.

Although overall alpha and beta diversity did not differ significantly with respect to UOG status, multiple OTUs were significantly correlated with the number of wells drilled in a given watershed after CSS normalization (**Figure 2**). Several of these taxa were also enriched based on UOG status (**Figure 3** and Supplementary Figure 7). LEfSe analysis revealed 34 enriched taxa for UOG- sites and 22 for UOG+ sites (LEfSe, α = 0.05; LDA score ≥ 2). EB1003, one of the taxa enriched in UOG- samples, had a negative correlation with number of wells drilled (Spearman, *R* = -0.4330, *p* = 0.0306) and conductivity (Spearman, *R* = -0.3842, *p* = 0.0435), while Flammeovirgaceae and Shewanellaceae, two of the taxa enriched in UOG+ samples, had positive correlations with conductivity (Spearman, *R* = 0.4857 and 0.4128, *p* = 0.0102 and 0.0324). Leptospirillaceae, Methanobacteriaceae, Rubrobacteraceae, and Shewanellaceae were enriched in UOG+ samples (**Figure 3**) and had positive correlations with the number of wells (Supplementary Table 4). LEfSe analysis also revealed that there are significant differences between the communities of UOG+ and UOG- samples even though they do not differ significantly in overall alpha and beta diversity. Furthermore, using a table with CSS normalized relative abundances of these enriched taxa, our random forest model had an average accuracy of 82.025% when classifying samples as UOG+/-. Methanobacteriaceae and EB1003 were especially important predictors, as they had the first and second greatest mean GINI decreases (1.1907 and 0.7787). However, it should be noted that some bacteria enriched based on UOG drilling presence were also enriched based on HUC10 watershed groupings (Supplementary Figure 8). A total of twelve bacterial families were significantly correlated with percent of woody wetland vegetation as well (Supplementary Table 5). Percent of woody wetland vegetation correlated with conductivity, and six of the twelve enriched bacterial families were also correlated with conductivity, including Shewanellaceae and EB1003, possibly suggesting the differences in their abundance may have been driven mainly by conductivity. Still, given these shared correlations, it is difficult to determine which factor had the greater effect on their abundances. Regardless, Shewanellaceae and EB1003’s correlations with the number of wells indicate that their abundance was affected, at least in part, by the presence of hydraulic fracturing. Moreover, when clustering was examined using PLS-DA, bacterial communities were determined not to cluster by HUC10 watershed (Supplementary Figure 5).

**FIGURE 4:**
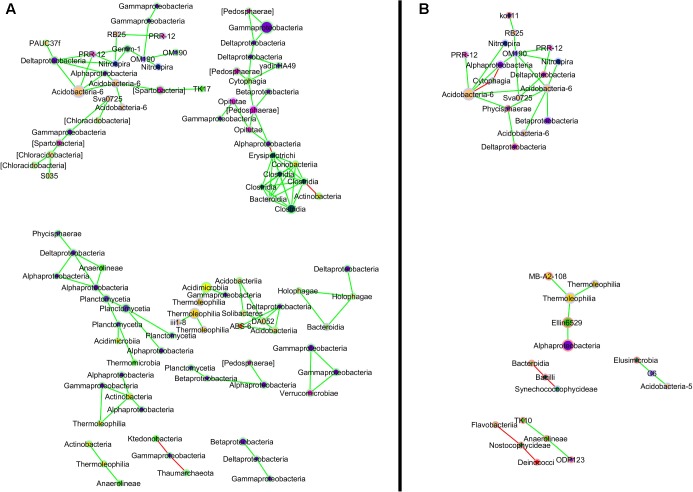
Co-occurrence network for UOG– **(A)** and UOG+ **(B)** sites. Nodes for OTUs exclusive to UOG– sites have a green border, and nodes for OTUs exclusive to UOG+ sites have a red border. OTUs present in both have a grey border. Nodes are labeled by class and colored by phylum; their size reflects their relative abundance, with larger nodes representing more abundant taxa. Pairs of nodes that were only connected to each other were excluded from the comparison network. See Supplemental **Figure 2** for the unaltered network.

In addition to changes in abundance of certain OTUs, interactions between OTUs also differed based on UOG drilling presence (**Figure 4**). The UOG- network had more nodes (149) and edges (138) than the UOG+ network (58 nodes and 45 edges). Most nodes were exclusive to one network. For example, Caulobacteraceae, Flammeovirgaceae, and Nostocaceae were only present in the UOG+ network, and Acetobacteraceae, Pedosphaeraceae, and Solibacteraceae were only present in the UOG- network. The UOG- network had a clustering coefficient of 0.193, a network centralization of 0.035, and a network density of 0.013. The UOG+ network had a lower clustering coefficient (0.052) but a higher network centralization (0.081) and network density (0.027). Altogether, the network modeling of these stream sediments indicates a more connected and robust bacterial community in UOG- samples as compared to UOG+ samples.

**FIGURE 5:**
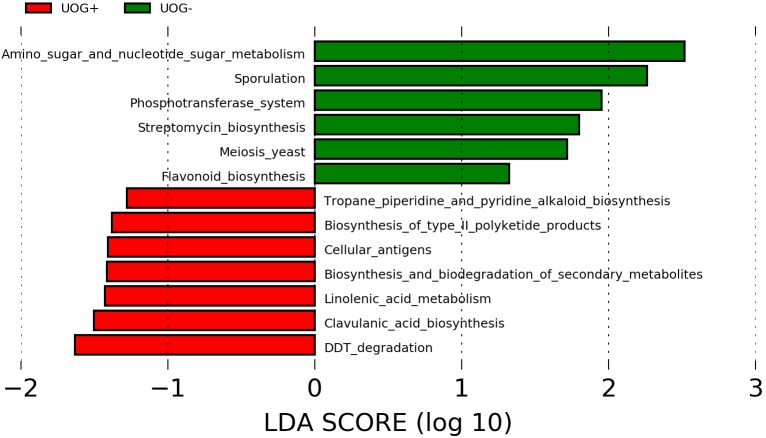
Predictive metagenomics using PICRUSt analysis. The functional capabilities of the bacterial communities in our samples were predicted with PICRUSt. LEfSe analysis was utilized to identify potentially enriched pathways. Pathways with a LDA score ≥1 are shown.

Metagenome functional content was predicted from 16S rRNA marker gene sequences using PICRUSt. The predicted functional underpinnings of UOG+ and UOG- stream sediment bacterial communities differs, as several predicted pathways were found to be enriched based on PICRUSt’s predictions (**Figure 5**). Using PICRUSt’s predictions, LEfSe identified six enriched pathways for UOG- and seven for UOG+ samples (LEfSe, α = 0.05; LDA score ≥ 1). Therefore, the prevalence of these pathways may have changed in response to hydraulic fracturing, likely being driven by the enrichment of certain bacteria. Within the enriched biosynthesis and biodegradation of secondary metabolites pathway, the genes K06219, K10531, K08977, K03932, K01865, and K00675 were more abundant in UOG+ samples than UOG- samples. Likewise, the enrichment of the linolenic acid metabolism pathway was driven largely by K00232 and K01058 and the enrichment of the clavulanic acid metabolism pathway was driven by K12675. Our NSTI values indicate the database was able to assign taxonomy to at least the order level accuracy, as they ranged from 0.1279 to 0.2040 for our samples (Supplementary Table 6).

**FIGURE 6:**
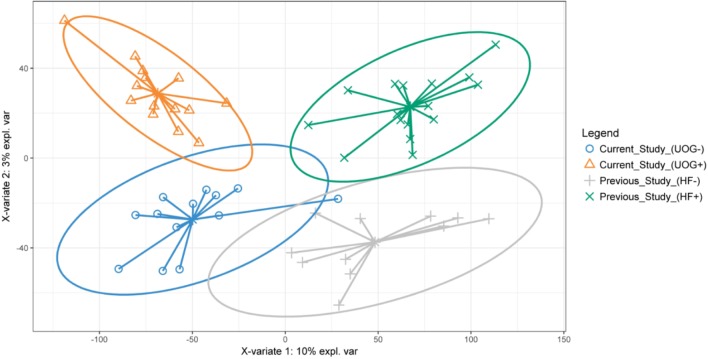
PLS-DA of stream sediments collected from our study (northeastern PA) and a previous study conducted in central PA. The PLS-DA was constructed using CSS normalized relative abundances of family level OTUs. Ellipses show the area where there is a 95% chance that samples in the group will be found. PLS-DA analysis was performed in R, using the mixOmics package (Cao et al., 2017).

PLS-DA revealed clustering based on potential geographic differences in bacterial community structure along the X-variate 1 axis, while samples appeared to be differentiated along the X-variate 2 axis based on the presence of hydraulic fracturing (**Figure 6**). In addition, there was more observed overlap in aquatic bacterial communities in non-impacted samples as compared to aquatic bacterial communities from streams impacted by hydraulic fracturing. A comparison of LEfSe analysis indicated some enriched taxa were present in both datasets. PAUC37f, EB1003, MND1, RB41, iii1_15, Nitrospiraceae, CCU21, and A21b were enriched in streams not near hydraulic fracturing for both studies while Methanobacteriaceae were enriched in streams near hydraulic fracturing for both studies (Supplementary Figures 3, 7). The consistent enrichment of these bacteria indicates they may be strongly impacted by the presence of hydraulic fracturing.

## Discussion

Here we assessed the potential impacts of UOG development on stream sediment bacterial communities in 28 streams in northeastern PA. To that end, data from high throughput sequencing of the 16S rRNA gene was used to compare bacterial communities in UOG+ and UOG- samples. Water quality data and 16S rRNA gene datasets were integrated to better understand how abiotic factors might correlate to shifts in bacterial communities within UOG+ and UOG- stream sediments. Deep sequencing coverage revealed diverse bacterial communities existed in both UOG+ and UOG- streams, as samples in both classifications had an average of over 3,000 different OTUs present. Interestingly, several bacterial assemblages differentiated UOG+ sites from UOG- sites. Furthermore, co-occurrence network models revealed a more robust and connected bacterial ecosystem within UOG- stream sediments, as compared to the disjointed bacterial community within the UOG+ stream sediment network (**Figure 5**), suggesting the sediment bacterial communities in those streams may have been disturbed. Previous literature has indicated land disturbances decrease the number of correlations among bacteria (Eldridge et al., 2015; Sun et al., 2017).

There were no significant differences in alpha diversity (Chao1, Heip’s evenness, observed, and PD whole tree) between UOG+ and UOG- streams assessed in this study, which is consistent with Johnson et al. (2017), in which there was no association between alpha diversity (Chao1, observed, Shannon evenness, and Shannon Entropy) and proximity to hydraulic fracturing activity. Trexler et al. (2014) observed a significant reduction in alpha diversity (Chao1, Heip’s evenness, observed, and PD whole tree) and pH in streams near Marcellus shale activity (UOG development). The decreased levels of alpha diversity in streams proximal to hydraulic fracturing may have been driven by pH, which is one of the main determinants of bacterial community composition (Dean et al., 2016). It should be noted that pH explained much less of the variation in bacterial community structure of streams assessed in this study as compared to Trexler et al. (2014).

While no measured water quality parameters were statistically significantly different between UOG+ and UOG- streams, we observed that samples with the highest conductivities were from UOG+ streams. This finding is consistent with Johnson et al. (2017); in which they report streams near hydraulic fracturing had higher conductivities than the streams not adjacent to hydraulic fracturing. Because flowback and produced water are typically saline (Olmstead et al., 2013; Lautz et al., 2014; Mouser et al., 2016), it would be expected that streams near UOG extraction could be more saline if they are being impacted by these fluids. An increase in water salinity would likely lead to an increase in salinity in underlying sediments, which would absorb the additional salts in the overlaying water. Shifts in the abundances of several bacterial populations have been observed in response to small increases in salinity (Wu et al., 2006; Campbell and Kirchman, 2013; Klier et al., 2018). Furthermore, recent work has shown that halotolerant and halophilic OTUs have been found to be highly abundant in fracking fluids (Mouser et al., 2016).

Stream conductivity significantly positively correlated with 61 different bacterial families, many of which are enriched in UOG+ samples and can live in saline conditions. For example, Cyclobacteriaceae has been successfully cultured on saline media, indicating it is at least halotolerant (Remmas et al., 2017). Members of the Rubrobacteraceae have been observed to be halophilic, as they have been found in relatively high abundances in samples derived from saline sediment (Kutovaya et al., 2015). Acidaminobacteraceae, Flammeovirgaceae, Methanobacteriaceae, Oceanospirillaceae, Shewanellaceae include bacteria found in marine environments (Cho et al., 2004; Mason et al., 2012; Bendall et al., 2013; Koo et al., 2015; Campeão et al., 2017; Dong et al., 2017), showing they can tolerate and thrive in high salinity environments. Furthermore, several enriched UOG+ OTUs included taxa encompassing known halophilic hydrocarbon degraders, such as Cyclobacteriaceae (Wang et al., 2014), Oceanospirallaceae (Lofthus et al., 2018), and Shewanellaceae (Bayat et al., 2015). Overall, the increased abundance of several halophilic taxa in UOG+ samples (Supplementary Figure 9) potentially suggests those streams may have been impacted by hydraulic fracturing. In order to determine a more definitive linkage, future work should include studies that investigate measurements both pre- and post-UOG development. Moreover, additional chemical measurements (e.g., ^87^S/^86^S isotopic ratios) of source fluids and stream samples would aid in classifying streams as impacted.

Increased concentrations of methane could potentially be indicative of streams impacted by hydraulic fracturing, as methane can enter underground water bodies due to hydraulic fracturing (Jackson et al., 2014; Burden et al., 2016) and can eventually contaminate surface water, potentially enriching taxa that use it as a carbon source. Two bacterial OTUs enriched in UOG+ sediments, Ignavibacteriaceae and Methylococcales, can both utilize methane (Wattam et al., 2014; Hestetun et al., 2016). Enrichment of these methanotrophs, could suggest these taxa are responding to increased methane concentrations within these samples. However, methane gases were not directly measured in these samples. Consequently, enrichment of these methanotrophs does not definitively indicate the streams have been impacted by methane as a result of hydraulic fracturing. Future work should directly measure methane gas within aquatic samples and use stable isotope probing, to determine if the source of this methane is from natural gas stores.

The enrichment of several different bacterial assemblages (i.e., halophiles, hydrocarbon degraders, methanotrophs, etc) in addition to the results of a more accurate random forest model, indicates these OTUs could serve as biomarkers for streams impacted by hydraulic fracturing. Interestingly, the three most important predictors for our model (Supplementary Figure 10) were all significantly correlated with the number of wells developed within a given watershed (**Figure 2**), suggesting they may be especially good indicators for strongly impacted streams. Our random forest results also suggest examining the abundances of taxa that differentiate the presence of hydraulic fracturing could serve as accurate indicators for streams impacted by hydraulic fracturing, however, the geo-spatial impacts on aquatic bacterial communities must be considered. PLS-DA of bacterial communities from this study and a previous study performed in central PA revealed that geographical location drives some of the differences observed in bacterial community structure (**Figure 6**). However, it was also observed that UOG+ (our study) and HF+ (previous study) bacterial communities differentiated from the more overlapping UOG-/HF- bacterial communities. Altogether, comparing these two sets of streams indicates that geographical factors may influence how hydraulic fracturing impacts nearby streams. Thus, future work is necessary to better understand the spatial and temporal stability of these potential biomarkers and their predictive power in correctly identifying other streams impacted by hydraulic fracturing activities.

Functional metagenomic predictions based on the 16S rRNA gene data provided additional evidence of the projected metabolic response of aquatic bacterial communities to potential hydraulic fracturing inputs. For example, genes involved in mechanisms of antibiotic resistance were enriched in UOG+ sites (K01058 and K12675). More specifically, genes within the UOG+ enriched linolenic metabolism pathway enable bacteria to resist antibiotics by reducing membrane permeability (Pedrotta and Witholt, 1999). Membrane permeability and efflux pumps have been shown, in the context of hydraulic fracturing, to be involved in a functional response to biocide compounds (Vikram et al., 2014, 2015). Biocides in hydraulic fracturing are often used at sublethal concentrations, which has been shown to select for resistance to these biocides, as multiple studies show diverse and active microbial communities associated with fracking-related fluids (Struchtemeyer and Elshahed, 2012; Vikram et al., 2016). The enrichment of these pathways could be a hallmark of the bacterial community responding to higher osmotic stress in UOG+ waters. Predictive metagenomics analysis also revealed the UOG+ bacterial communities were enriched in functions mapping to biodegradation pathways. For example, benzene (K10531) and nitrobenzene (K01865) degradation were enriched in UOG+ samples. Interestingly, benzene has been found in produced waters (Burden et al., 2016). Although predictive metagenomics was useful in generating some mechanistic hypotheses about the role of enriched taxa in this environmental scenario, tools like PICRUSt are constrained by reference databases. Accordingly, future work should utilize shotgun metagenomics and metatranscriptomics to quantify genetic potential and expression of microbial communities under varied hydraulic fracturing impacts.

Altogether, our study revealed that despite similar overall bacterial diversity between UOG+ and UOG- communities, UOG drilling status was associated with differential bacterial assemblages within surrounding stream ecosystems. Most notably, stream sediment bacterial communities in UOG+ streams were enriched in methanotrophic, salt-tolerant, and hydrocarbon-degrading taxa. Our random forest model indicated several of these enriched taxa could be effective biomarkers for determining if a stream has been impacted by hydraulic fracturing, and future longitudinal studies could even shed light on the natural attenuation and recovery of these ecosystems. Furthermore, our data suggest certain metabolic pathways could be indicative of impacts associated with hydraulic fracturing in streams in close proximity to UOG extraction. Future utilization of matched metagenomics and metatranscriptomics sequencing will provide a more robust understanding of the functional response of bacterial communities to hydraulic fracturing impacts on proximal streams. Additionally, future studies should include samples from hydraulic fracturing flowback and produced water and characteristic chemical properties of that wastewater to better establish a link between hydraulic fracturing and its direct impact on proximal streams.

Unfortunately, confounding factors due to differences between streams unrelated to hydraulic fracturing, such as the amount of organic matter in the streams or the influence of other anthropogenic factors due to runoff, such as road salts, cannot be completely ruled out and could have impacted our results. Thus, microcosm studies under controlled conditions, similar to Campa et al. (2018), could also be useful to pinpoint hydraulic fracturing’s impact on aquatic microbial communities. Despite these limitations, our study contributes to the growing knowledge about hydraulic fracturing’s impact on the environment and highlights the need for additional research to further understand its potential environmental impacts.

## Author Contributions

RL, JN, and CG designed the study with input from TH and MC. JN and CG collected the samples. CM, NU, HN, and JCS processed the samples for sequencing. JCS and HN analyzed the data with help from CM, NU, JW, and VT. JCS, RL, and HN wrote the manuscript with help from MC, CG, JN, and TH. DR performed in depth GIS and statistical analyses for the manuscript.

## Conflict of Interest Statement

The authors declare that the research was conducted in the absence of any commercial or financial relationships that could be construed as a potential conflict of interest.
