# Supplementary material for: Bacterial Biomarkers of Marcellus Shale Activity in Pennsylvania
Source: Front Microbiol. 2018 Aug 2;9:1697. doi: 10.3389/fmicb.2018.01697 (PMC6083035; doi:10.3389/fmicb.2018.01697)
Supplement: Supplementary file 7 [file Data_Sheet_1.docx]

Supplementary Material

**Bacterial Biomarkers of Marcellus Shale Activity in Pennsylvania**

Jeremy R. Chen See^1^, Nikea Ulrich^1^, Hephzibah Nwanosike^1^, Christopher J. McLimans^1^, Vasily Tokarev^1^, Justin R. Wright^1^, Maria F. Campa^2^, Christopher J. Grant^1^, Terry C. Hazen^2,3,4^, Jonathan M. Niles^5^, Daniel Ressler^6^, Regina Lamendella^1*^

^1^Juniata College, Department of Biology, Huntingdon, PA, USA

^2^ Bredesen Center, University of Tennessee, Knoxville, TN 37996, USA

^3^ Department of Civil and Environmental Engineering, University of Tennessee, Knoxville, TN 37996, USA

^4^ Biosciences Division, Oak Ridge National Laboratory, Oak Ridge, TN 37831, USA

^5^ Freshwater Research Initiative, Susquehanna University, Selinsgrove, PA, 17870 USA

^6^ Susquehanna University, Department of Earth and Environmental Sciences, Selinsgrove, PA, 17870 USA

*** Correspondence:**

Corresponding Author: Regina Lamendella

lamendella@juniata.edu


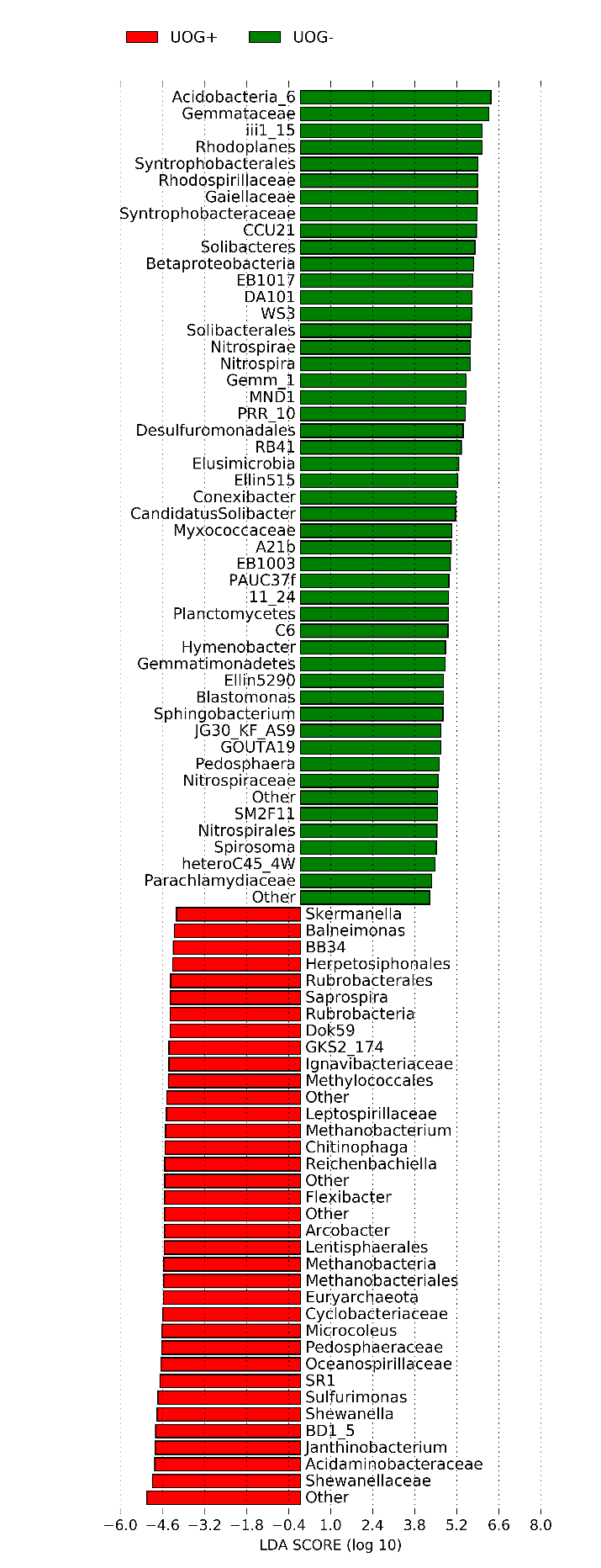


**Supplemental Figure 1**. Significantly enriched taxa based on the presence of hydraulic fracturing. Taxa were identified using LEfSe with CSS normalized relative abundances at the genus level. The green bars show taxa that are more common in UOG- samples, and the red bars show taxa that are more common in UOG+ samples. Enrichment is shown on a log scale. LEfSe compares classes using a Kruskal Wallis test (α ≤ 0.05) to determine which taxa are differentially enriched. Only enriched OTUs with a LDA score ≥ 2 are shown. Relative abundances from a CSS normalized family level OTU table were used as input.


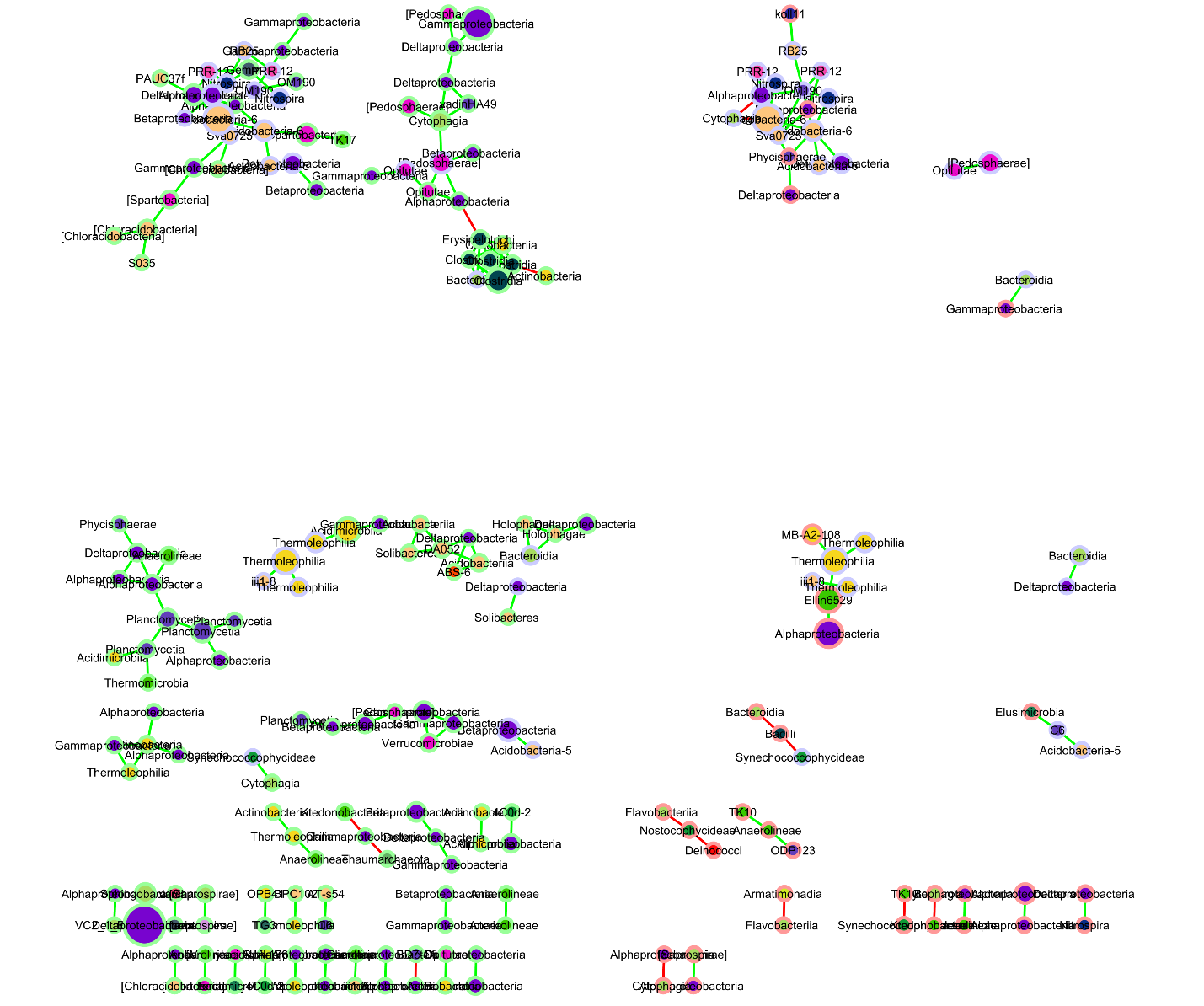


**Supplemental Figure 2**. Unmodified co-occurrence network for UOG- (left) and UOG+ (right) sites. Nodes for OTUs exclusive to UOG- sites have a green border, and nodes for OTUs exclusive to UOG+ sites have a red border. OTUs present in both have a grey border. Nodes are labelled by class and colored by phylum; their size reflects their relative abundance with more abundant taxa being represented by larger nodes.


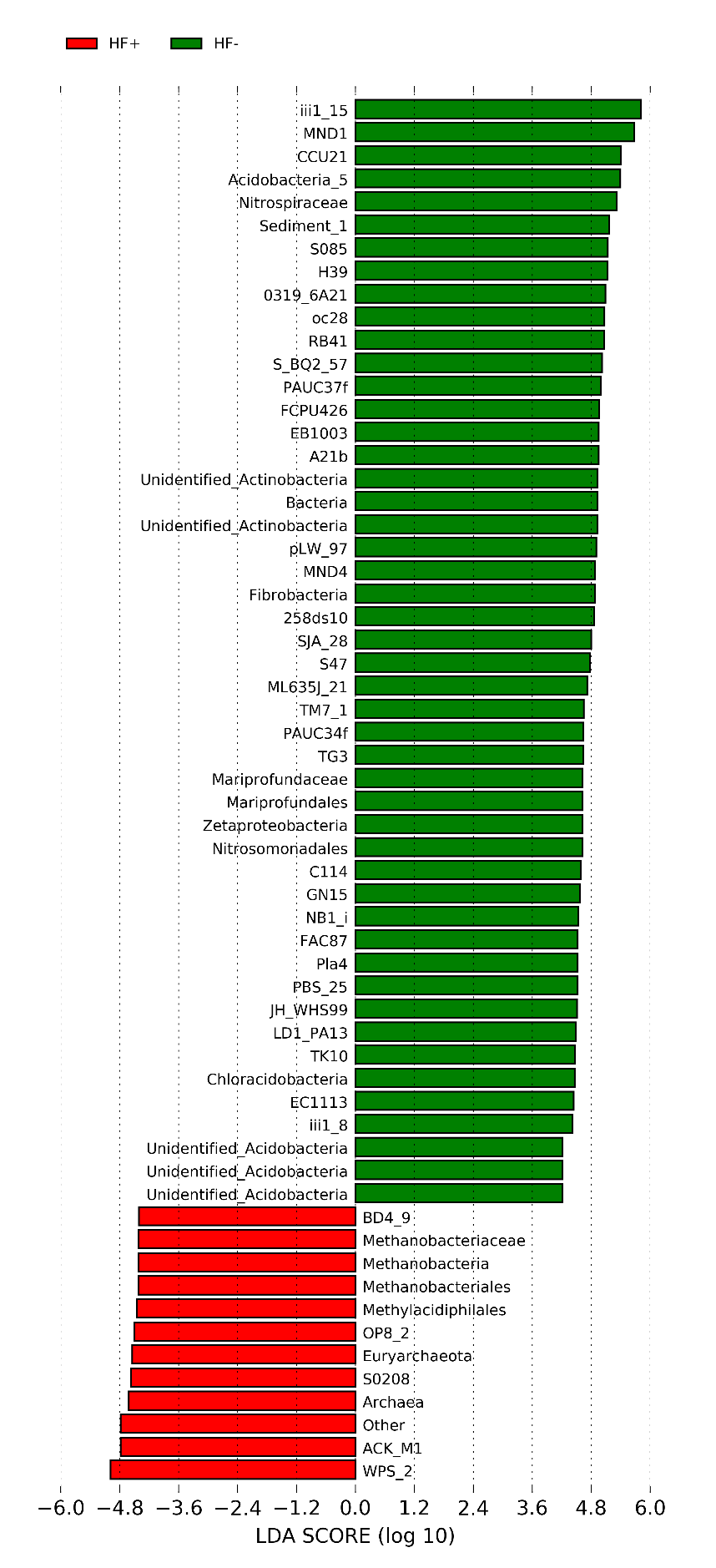


**Supplemental Figure 3**. Significantly enriched taxa in the 2014 sediment samples based on presence of hydraulic fracturing. Taxa were identified using LEfSe as previously described with an OTU table containing relative abundances based on CSS normalized data at the family level.

**Supplemental Figure 4**. PLS-DA script for R using mixOmics package.


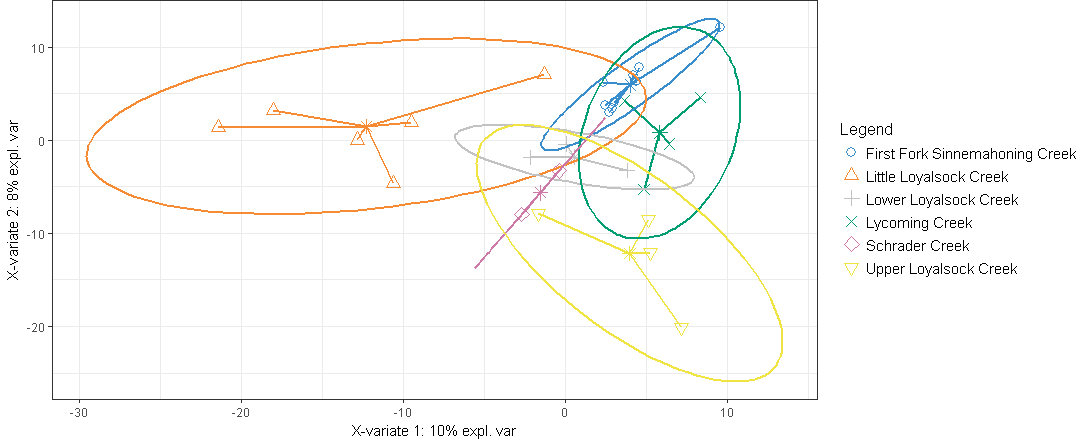


**Supplemental Figure 5**. PLS-DA for samples grouped by HUC10 watershed. The PLS-DA was constructed using CSS normalized relative abundances of family level OTUs. Ellipses show the area where there is a 95% chance that samples in the group will be found. PLS-DA analysis was performed in R, using the mixOmics package (Cao et al., 2017).


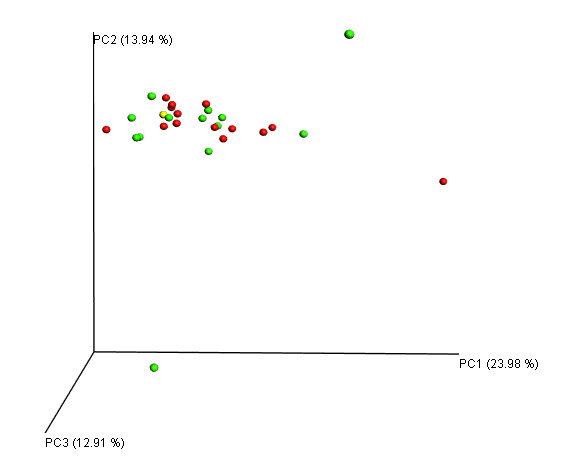


**Supplemental Figure 6**. PCoA of sampling sites based on UOG drilling created using EMPeror (Vázquez-Baeza et al., 2013). UOG- samples are green, UOG+ samples are red, and the UOG abandoned sample is yellow. A weighted unifrac distance matrix was used.


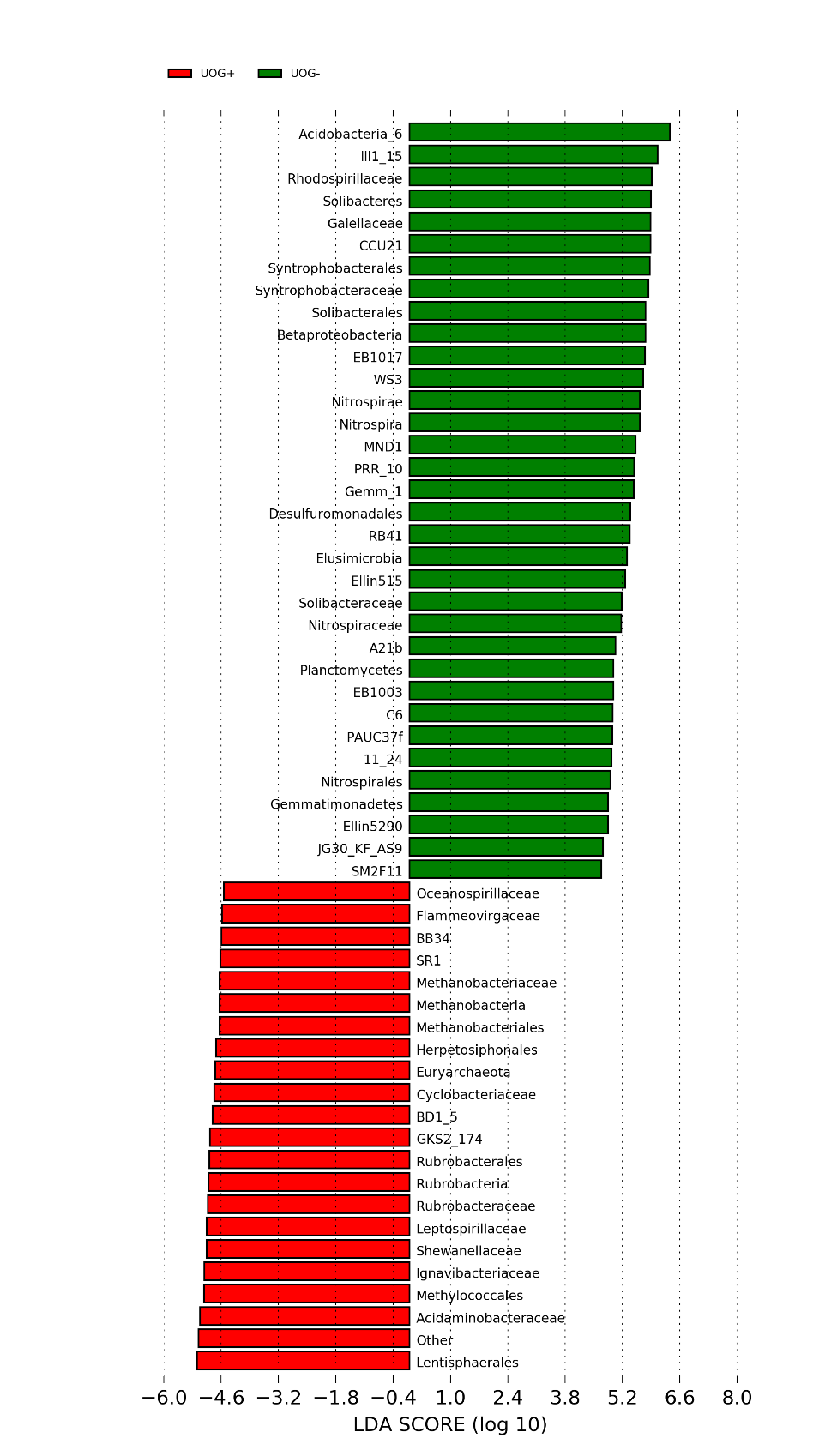


**Supplemental Figure 7**. Significantly enriched taxa in UOG+ and UOG- samples. Taxa were identified using LEfSe as previously described with an OTU table containing relative abundances based on CSS normalized data at the family level.


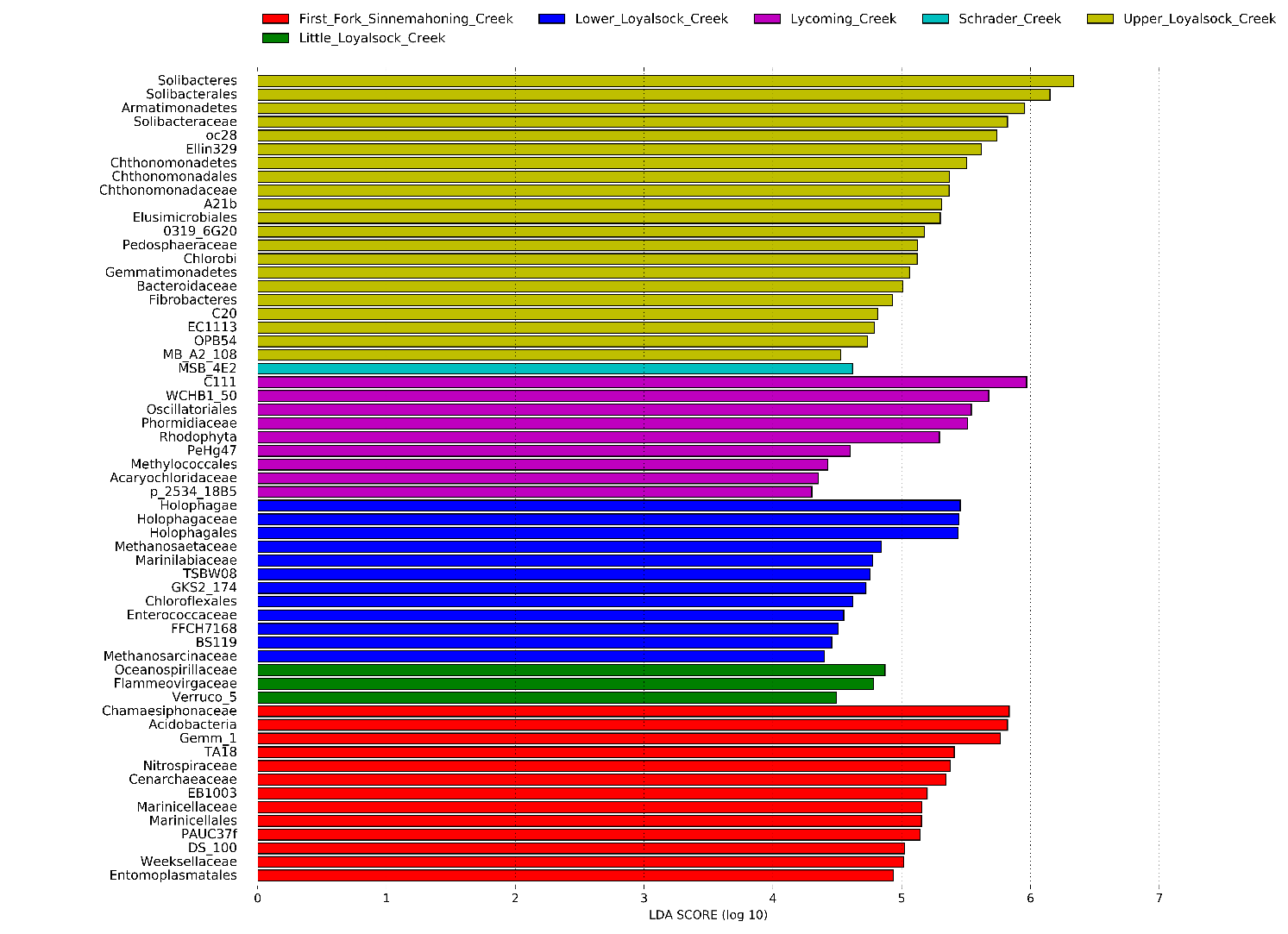


**Supplemental Figure 8**. Significantly enriched taxa based on HUC10 watershed. Taxa were identified using LEfSe as previously described. PAUC37f, EB1003, Nitrospiraceae, Gemm_1, Oceanospirillaceae, Flammeovirgaceae, GKS2_174, Methylococcales, Solibacteres, Solibacteraceae, Solibacterales, A21b, and Gemmatimonadetes are enriched based on both HUC10 watershed and UOG drilling presence, suggesting geography could be a confounding factor. Notably, Flammeovirgaceae and Oceanospirillaceae are both enriched in the watershed containing streams with the highest conductivities.


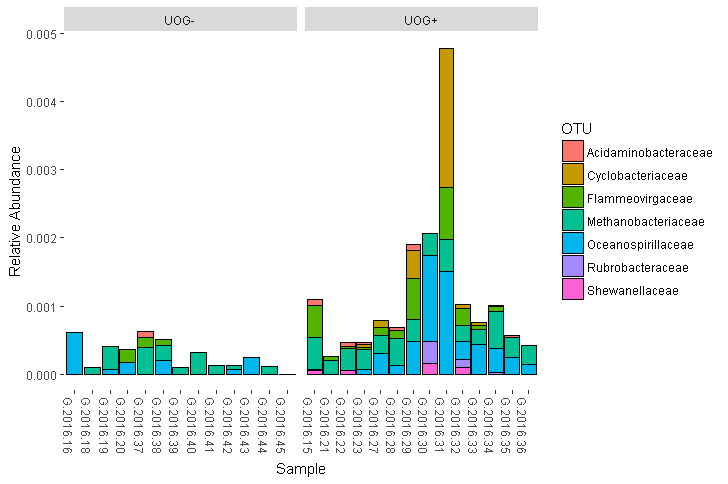


**Supplemental Figure 9**. Stacked bar plot of CSS normalized relative abundances for enriched families that have been found to be salt tolerant.


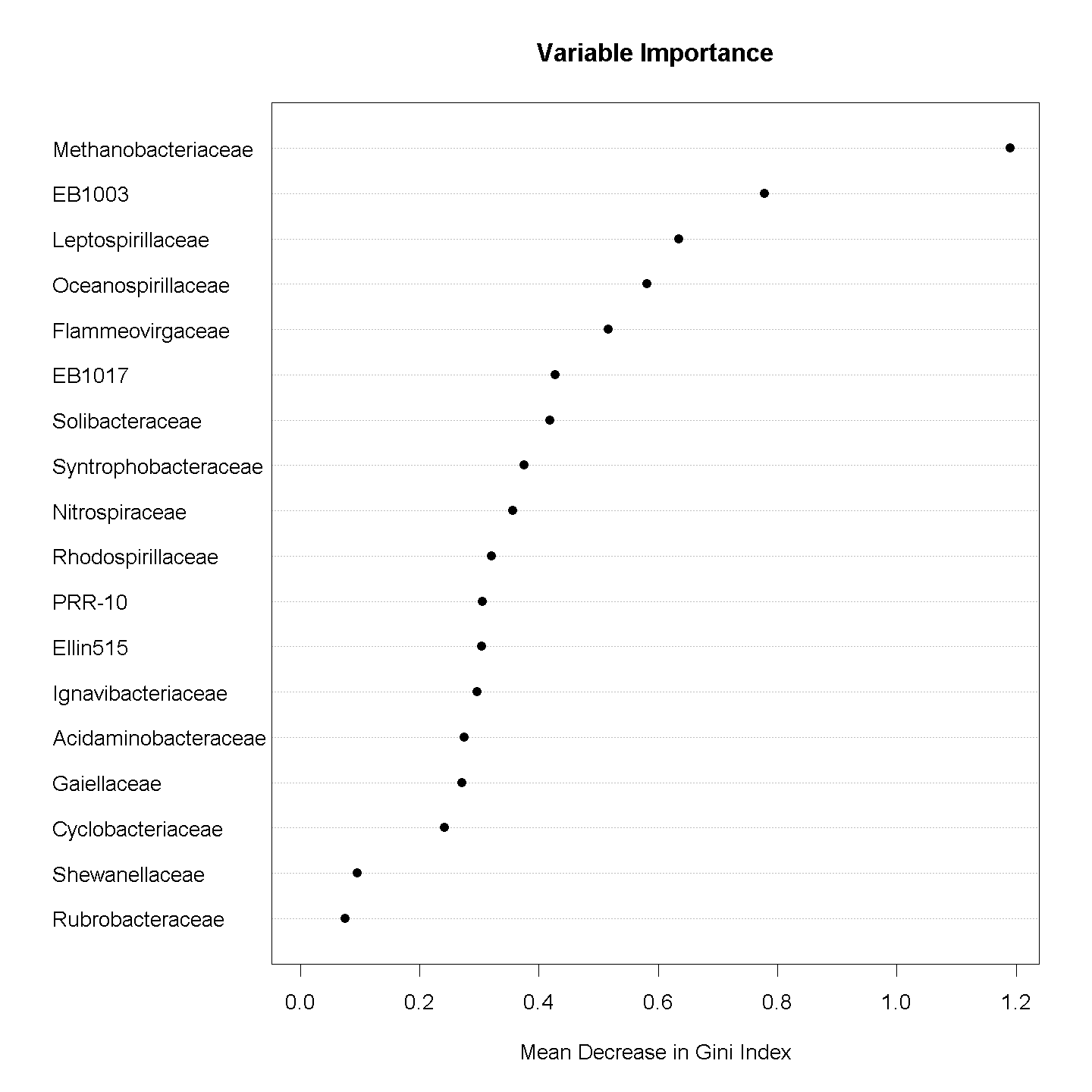


**Supplemental Figure 10**. Variable importance plot for the classification of sites as UOG+ or UOG- using a random forest model based on the relative abundances of enriched OTUs.  The y axis shows the OTUs, and the x axis shows their mean decrease in Gini index. Taxa with a higher mean decrease were more important for classifying the sites.
